# Acid secretion by the boring organ of the burrowing giant clam, *Tridacna crocea*

**DOI:** 10.1098/rsbl.2018.0047

**Published:** 2018-06-13

**Authors:** Richard W. Hill, Eric J. Armstrong, Kazuo Inaba, Masaya Morita, Martin Tresguerres, Jonathon H. Stillman, Jinae N. Roa, Garfield T. Kwan

**Affiliations:** 1 Department of Integrative Biology, Michigan State University, East Lansing, MI 48824, USA; 2 Department of Integrative Biology, University of California, Berkeley, CA 94720, USA; 3 Estuary and Ocean Science Center, San Francisco State University, Tiburon, CA 94920, USA; 4 Shimoda Center, University of Tsukuba, Shimoda, Shizuoka 4150025, Japan; 5 Sesoko Station, University of the Ryukyus, Motobu, Japan; 6 Scripps Institution of Oceanography, University of California, La Jolla, CA 92093, USA

**Keywords:** bivalve, pH, bioerosion, vacuolar-type H^+^-ATPase

## Abstract

The giant clam *Tridacna crocea*, native to Indo-Pacific coral reefs, is noted for its unique ability to bore fully into coral rock and is a major agent of reef bioerosion. However, *T. crocea*'s mechanism of boring has remained a mystery despite decades of research. By exploiting a new, two-dimensional pH-sensing technology and manipulating clams to press their presumptive boring tissue (the pedal mantle) against pH-sensing foils, we show that this tissue lowers the pH of surfaces it contacts by greater than or equal to 2 pH units below seawater pH day and night. Acid secretion is likely mediated by vacuolar-type H^+^-ATPase, which we demonstrate (by immunofluorescence) is abundant in the pedal mantle outer epithelium. Our discovery of acid secretion solves this decades-old mystery and reveals that, during bioerosion, *T. crocea* can liberate reef constituents directly to the soluble phase, rather than producing sediment alone as earlier assumed.

## 1. Introduction

**Figure 1.**
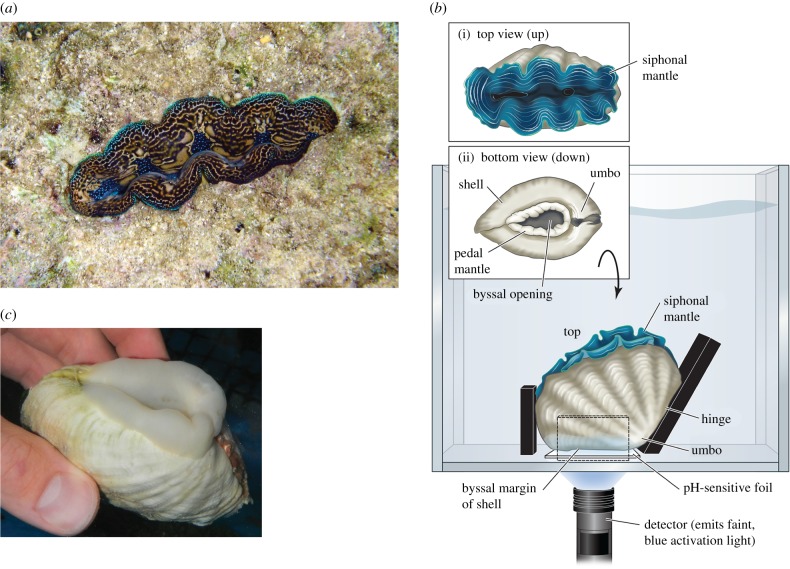
(*a*) *Tridacna crocea* in the wild, viewed from above (courtesy of James Fatherree). The siphonal mantle is expanded above the surrounding coral rock. (*b*) Clam in open-topped study aquarium. (*c*) Clam suddenly removed from coral rubble and photographed immediately, showing the expanded pedal mantle protruding from the byssal opening (compare *b*(ii)). (Online version in colour.)

*Tridacna crocea*—abundant in many Indo-Pacific coral reef communities—is in certain ways the most remarkable of the giant (tridacnid) clams. Like all tridacnids, it has a symbiotic relationship with dinoflagellate algae, which photosynthetically produce much of its energy substrates. The algal cells live within the *siphonal mantle*, a tissue that the clam presents to the sun as a light antenna. *Tridacna crocea* is unique among tridacnids in that it bores fully into the coral rock (the solid skeletal material formed by massively growing corals). It expands its siphonal mantle above the rock surface (figure 1(*a*)) with no visible sign of the rest of its body, which is within a chamber bored in the rock by the clam [1]. If threatened, the clam withdraws its siphonal mantle so that its entire body is inside the rock, thereby fully exploiting the rock as extra-somatic armour. Each individual starts boring early in life, enlarging its chamber as it grows. Full-grown individuals (10–14 cm long), 30–80 years old on the Great Barrier Reef [2], remain fully ensconced in the rock. Ecologically, *T. crocea* can be exceedingly numerous (more than 100 individuals m^−2^) [2].

*Tridacna crocea*'s mechanism of boring has long been pondered by biologists, most notably Sir C. M. Yonge. Boring by acid secretion has been hypothesized often because coral rock is a type of limestone, subject to acid dissolution. As early as 1936, Yonge [3] added pH indicator to seawater bathing the presumptive boring organ but found no evidence for acid secretion. In fact, acid secretion has never been demonstrated (as fully discussed in the electronic supplementary material), for reasons we believe we now understand (see Discussion). Five decades after his initial experiments, Yonge [1] summarized a lifetime of work, unable to point to documented mechanisms but hypothesizing that *T. crocea* bores by both mechanical rasping and non-acid chemical boring.

Knowledge of *T. crocea*'s boring mechanism is ecologically and evolutionarily important for several reasons. First, *T. crocea* is one of the largest animals that can bore completely into rock, and its abundance and size make it a key contributor to reef bioerosion [2]. Second, the immediate products of *T. crocea*'s bioerosion are determined by the mechanism it uses to bore. Third, recent research points to a dramatic evolutionary convergence among diverse tissues and animals in the mechanism employed to dissolve carbonate substrates, namely H^+^ secretion mediated by vacuolar-type H^+^-ATPase (VHA) [4]. Establishing the phylogenetic extent of this convergence is of high interest.

Here we definitively address the question of acid secretion by *T. crocea*. By exploiting a new technology and carefully considering the morphology and behaviour of *T. crocea*, we manipulated clams so that they pressed their presumptive boring tissue against two-dimensional, pH-sensitive foils. We also used immunohistochemical methods to examine the presence and localization of VHA in the boring epithelium.

## 2. Relevant morphology

In all tridacnid species, the umbo—the central meeting point of the two valves (half shells)—is positioned down (figure 1*b*). Thus the siphonal mantle—located between the valve margins distal to the umbo—faces up and is spread out in the sun's rays when the giant clam gapes (figure 1*b*(i)). *Tridacna crocea* is distinctive in the morphology of its underside, where there is a large gap—the *byssal opening*—between the valve margins (figure 1*b*(ii)); although other tridacnids have this opening, it is largest in *T. crocea* [1]. The opening is surrounded by *pedal mantle* tissue, shown contracted (figure 1*b*(ii)). This soft tissue can be expanded enormously by inflation with blood and has long been considered the likely site of acid secretion if such secretion occurs [1,2].

## 3. Material and methods

### (a) Acquisition of *Tridacna crocea*

Studies were conducted (March 2017) at the Sesoko Station (University of Ryukyus), Okinawa, Japan. *Tridacna crocea* measuring 2–9 cm long were collected (under Item 3 of the Japanese law for the common right of fishery) by scuba from the offshore fringing reef, without cutting or tearing tissue, and housed on fine (approximately pea-size) coral rubble in an outdoor, flow-through seawater system. Water temperature was the same as the sea temperature where clams were collected (21°C). Lighting was entirely by sunlight. Studies were completed within 4 days after collection, during which all giant clams remained vigorously healthy.

### (b) Study of pedal mantle surface pH with pH-sensitive foils

Each clam used in this part of our research was studied (full details in the electronic supplementary material) in a small, open-topped, acrylic aquarium. The clam was kept in the desired orientation by individually positioned fence pieces (figure 1*b*): two end pieces (black)—one at a 120° angle to the bottom to match the angle formed by the shell at the umbo—and two side pieces (dashed). Nothing was attached to the clams, and they were free to open fully and extend the siphonal and pedal mantle, which they invariably did.

To measure pH, we used a new two-dimensional (2D) optrode technology (PreSens Precision Sensing). A thin pH-sensitive foil (PreSens SF-HP5R) was positioned on the acrylic substrate under the clam's byssal opening (figure 1*b*).

A key insight for our research design is that when a *T. crocea* is positioned as in figure 1(*b*), the clam's entire lower margin is nearly parallel with the substrate. Accordingly, when the pedal mantle is extended, it promptly encounters the substrate and presses against it, thereby—with the pH-sensitive foil in place—permitting sustained pH measurement at the tissue surface.

Nine clams of diverse sizes were studied in individual aquaria, where they lived continuously, day and night, submerged in the flow-through seawater system. The foil was installed in each aquarium when the clam was introduced and remained there continuously. For pH measurement, an aquarium was removed for approximately 10 min from the seawater system and positioned (figure 1*b*) above a PreSens detector for imaging of the foil from below (all images done in duplicate). After clams had been in the aquaria and seawater system for 24 h, the first images were acquired during daytime (13.30–15.20 Japan Standard Time (JST)), followed by second images at night (20. 40–22.30 JST).

Foils were calibrated using seven small foil pieces (smaller than foils but from same manufacturing batch) exposed to seven known-pH buffer solutions at the ionic strength of seawater (see the electronic supplementary material). The calibration pieces were imaged simultaneously with foils in aquaria.

### (c) Visualization of vacuolar-type H^+^-ATPase and study of pH with microprobes

After the foil study, pedal mantle was vivisected from five of the clams for investigation of VHA. Tissue fixed at Okinawa was transported to Scripps Institution of Oceanography (CITES permit 17JP003387/TE), where immunofluorescence staining was performed using VHA_B_-specific antibodies (details in the electronic supplementary material).

Tissue pH was also studied with PreSens fibre-optic optrodes mounted in long hypodermic needles (details in the electronic supplementary material). This type of optrode measures pH at a single point, its 0.14 mm-diameter tip. Our goal was to place the tip in contact with the pedal mantle, usually by inserting the tip under the mantle where it contacted the acrylic substrate. Four clams not used in the foil study were investigated.

## 4. Results

### (a) Study of pedal mantle surface pH with pH-sensitive foils

Giant clams housed on fine coral rubble in the seawater system extended their pedal mantle to the same extent as clams in aquaria. We photographed the shape and extent of the extended pedal mantle by removing clams suddenly from rubble (figure 1*c*). Ideally we would have photographed clams in aquaria from below as they were studied (figure 1*b*). However, the pH-sensitive foils are opaque.

**Figure 2.**
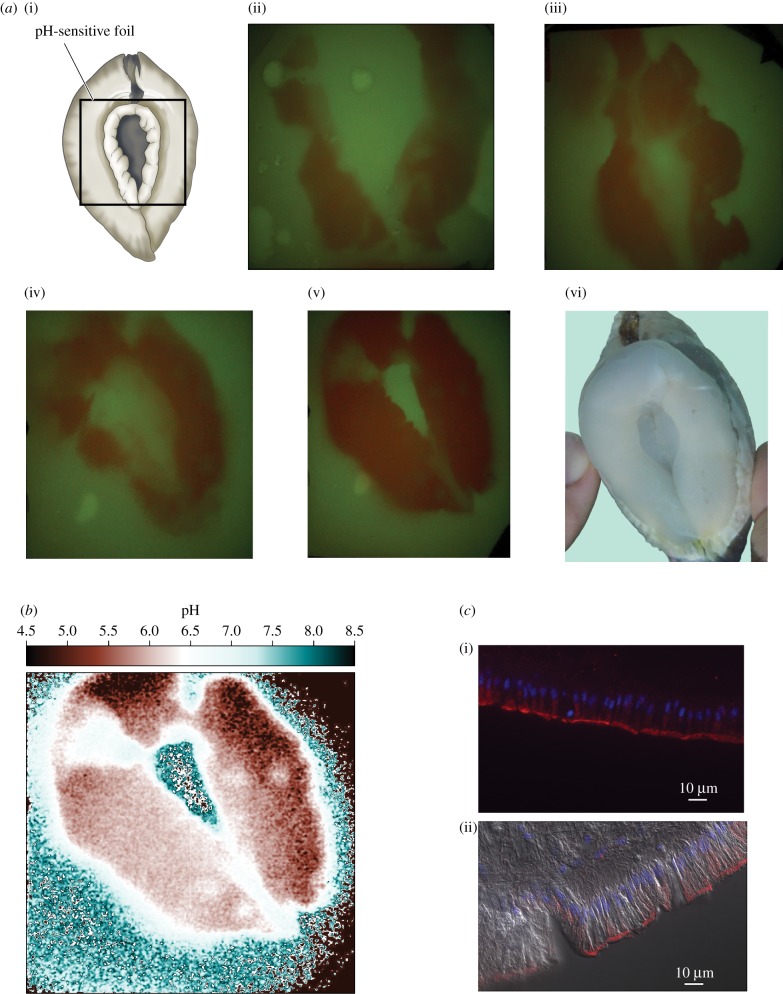
Results. (*a*(i)) Orientation of clam and foil in the study aquarium as viewed from below, i.e. as viewed by the detector. (*a*(ii–v)) Two-dimensional pH fields in pH-sensitive foils (green, alkaline; red, acidic) as viewed by the detector from below in the same orientation as in (*a*(i)). Images show three individuals: one during daytime (*a*(ii)), a second during nighttime (*a*(iii)) and a third daytime (*a*(iv)) and nighttime (*a*(v)). (*a*(vi)) Clam photographed as in figure 1*c* in the same orientation as in (*a*(i)). (*b*) pH-sensitive foil image calibrated on a pixel-by-pixel basis. (*c*) Immunolocalization of VHA (red) in sections of pedal mantle viewed by epifluorescence microscopy (cell nuclei stained blue with Hoescht 33342): (*c*(i)) VHA is abundant along the pedal mantle margin (facing down) which contacts substrate during boring. (*c*(ii)) VHA is overlapped here with a differential interference contrast image showing cuboidal morphology of VHA-rich epithelial cells. (Online version in colour.)

The 2D pH fields observed in the foils of three clams are shown in figure 2*a*(ii–v). In these images red represents relatively acidic (low) pH and green relatively alkaline (high) pH (mostly seawater pH, 8.2). Large regions of lowered pH are evident and were observed in all 18 foils imaged: 9 clams, daytime and nighttime. In each image, the average pH in an approximately 2 mm × 2 mm dark red region and in a randomly chosen approximately 2 mm × 2 mm peripheral green region were compared. Red areas are significantly more acidic than green (paired *t*-tests: d.f. = 8, *p* < 0.0001 for daytime images; d.f. = 8, *p* < 0.001 for nighttime).

Strikingly, the 2D shape of the low pH, red regions (figure 2*a*(ii–v)) resembles the 2D shape of the most extended bands of pedal mantle (figure 2*a*(vi); figure 1*c*). The low-pH regions are like a fingerprint of the pedal mantle.

The foils respond rapidly to changes of pH. When tested under the conditions used in this study (relatively still water; 21°C), foils attain 90% of full response to a change of 1 pH unit in either direction in ≤1 min (details in the electronic supplementary material). Thus a region of foil must be currently touched by a pH-lowering part of the pedal mantle to have a low pH, and pH values measured daytime and nighttime are independent.

Using calibrated images (figure 2*b*), we have tabulated quantitative data on foil acidification by the pedal mantle (table 1). According to two-tailed, paired *t*-tests, time of day has no significant effect on minimum pH (d.f. = 8, *p* = 0.25) or mean pH within a region of interest (ROI) surrounding the minimum (d.f. = 8, *p* = 0.67).

**Table 1.** Minimum pH and mean pH in a 1.96 × 1.96 mm region of interest (ROI) centred on the point of minimum pH. Mean ± 95% confidence intervals. Seawater pH was 8.2.

time	minimum pH	mean pH within ROI
daytime (*n* = 9)	5.36 ± 0.68	6.12 ± 0.19
nighttime (*n* = 9)	4.65 ± 1.2	6.05 ± 0.31

### (b) Vacuolar-type H^+^-ATPase

VHA is abundant on the apical microvilli of outer epithelial cells of *T. crocea* pedal mantle (figure 2*c*; controls discussed in the electronic supplementary material).

### (c) Study of pH with microprobes

For 4 h we attempted more than 100 times in four clams to place the pH-sensitive tip of a microprobe in contact with the pedal mantle. Measured pH always remained close to seawater pH. Using magnifying lenses, we saw that invariably the mantle contracted (usually locally and subtly) within 1–2 s when touched, pulling the mantle tissue out of contact with the probe tip and creating a seawater-filled space (often less than 1 mm) between tissue and probe.

## 5. Discussion

Yonge [1] and others [2] worked valiantly in the field to open *T. crocea* chambers in the coral rock of natural reefs. They reported that when the pedal mantle is hydraulically extended from the byssal opening, it often protrudes great distances, expanding up both sides of the giant clam [1] and sometimes reaching the top [2]. It thus can play a widespread role in shaping and enlarging a clam's chamber.

Our foil images (figure 2*a*(ii–v)) demonstrate that the pedal mantle (shaped as in figure 2*a*(vi)) dramatically lowers the pH of a surface where it makes contact—pointing to acid secretion by the tissue. Individual *T. crocea* of all sizes tested (2.3–8.9 cm long)—corresponding to ages of 1–10 years [2]—secrete acid for boring both day and night, lowering the pH of contacted surfaces by ≥2 pH units below seawater pH (table 1).

VHA is abundant in the outer tissue epithelium of pedal mantle pressed against the substrate, pointing to VHA-mediated H^+^ secretion as the mechanism of acidification. This finding contributes further evidence for the emerging paradigm that VHA-mediated H^+^ secretion is a pre-eminent mechanism of carbonate dissolution across a wide diversity of animal phyletic groups, ranging from *Osedax* bone-eating annelids to osteoclast cells of vertebrates [4].

Although the pedal mantle acidifies surfaces it contacts, our microprobe studies demonstrate that—at the scale of a 0.14 mm sensor—the pedal mantle does not acidify the juxtaposed seawater (see also figure 2*b*). A similar phenomenon was reported in early studies of oyster drills (gastropods) [5]. *Tridacna crocea*'s failure to acidify seawater explains why Yonge [3] did not detect acid secretion using pH indicator in seawater near the pedal mantle—a finding that led him and others to downplay the possibility of acid secretion for decades [1].

Our results demonstrate that bioerosion by *T. crocea* is in part chemically mediated by acid secretion. This discovery overturns the earlier assumption that bioerosion is entirely mechanical [2], forcing reconsideration of erosion dynamics. In the earlier view [2], the sole immediate product of bioerosion was pulverized reef rock, which awaited other processes to be recycled. With acid secretion being at least partly responsible for bioerosion, *T. crocea* can directly and immediately liberate reef constituents (e.g. Ca^2+^) to the soluble phase and alter 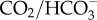 concentrations—thereby directly affecting calcification and photosynthesis on the reef (e.g. by corals and tridacnids).

## Supplementary Material

Supplements to the text of the manuscript

## Data accessibility

Data have been uploaded to Dryad: (http://dx.doi.org/10.5061/dryad.88s50) [6].
