# Supplementary material for: Acid secretion by the boring organ of the burrowing giant clam, Tridacna crocea
Source: Biol Lett. 2018 Jun 13;14(6):20180047. doi: 10.1098/rsbl.2018.0047 (PMC6030592; doi:10.1098/rsbl.2018.0047)
Supplement: Supplements to the text of the manuscript [file rsbl20180047supp1.docx]

**Acid secretion by the boring organ of the burrowing giant clam, *Tridacna crocea***

Richard W. Hill^1,*^, Eric J. Armstrong^2,3^, Kazuo Inaba^4^, Masaya Morita^5^, Martin Tresguerres^6^, Jonathon H. Stillman^2,3^, Jinae N. Roa^6^, and Garfield T. Kwan^6^

^1^Department of Integrative Biology, Michigan State University, East Lansing, MI 48824 USA

^2^Department of Integrative Biology, University of California, Berkeley, CA 94720 USA

^3^Estuary and Ocean Science Center, San Francisco State University, Tiburon, CA 94920 USA

^4^Shimoda Center, University of Tsukuba, Shimoda, Shizuoka 4150025, Japan

^5^Sesoko Station, University of the Ryukyus, Motobu, Japan

^6^ Scripps Institution of Oceanography, University of California, La Jolla, CA 92093 USA

^*^Corresponding author: hillr@msu.edu

**Supplementary Material**

**Important:** This ESM document has its own, complete list of literature citations, found at the end of the document. For reading the ESM, please use this ESM-specific list, not the References list in the published paper. Where this ESM refers to figure numbers, the figures are those in the published paper, except that Figure S1 is found only in the ESM.

**Supplementary Introduction**

***Evidence that prior claims of acid secretion were not supported by data***

Already in 1921 Hedley [1] expressed exasperation over instances in which biologists, with “much authority,” asserted attributes of tridacnids that were in fact not supported by data. The prior history of the acid secretion hypothesis is a case in point.

Hobbyists sometimes assert acid secretion simply because it seems logical or they have heard that it occurs from other hobbyists. For providing a citation in the scientific literature, the most common paper cited is Suzuki’s [2] . Suzuki stated simply that *T. crocea* “erodes the substrate with acid” during boring. She cited Hedley [1], Kawaguti [3], and Yonge [4] as authority for this statement. In fact, Hedley [1] did not discuss acid. Kawaguti [3], studying just the earliest developmental stages, stated that “acid glands” are present in the anterior mantle of early juvenile *T. crocea*, but he said nothing more; he apparently planned to publish evidence for his statement in the future, but never did, and in any case he made no statement about individuals other than early juveniles. As for Yonge [4], writing in his career-spanning, detailed overview of the Tridacnidae, he stated that “no secretion of acid has been detected by any worker.” One cannot imagine three more competent and esteemed biologists than Hedley, Kawaguti, and Yonge. Only one of them, however, believed he had evidence (unpublished and unstated) for acid secretion, and Yonge explicitly disavowed use of acid. Thus, Suzuki’s certain statement had no cited support that would justify certainty.

**Supplementary Material and Methods**

***Study of pedal mantle surface pH with pH-sensitive foils***

Each clam was studied in an individual, open-topped study aquarium, made of 0.54-cm-thick clear acrylic sheet, of standardized size (13 cm × 15 cm × 13 cm high). To restrict clam movements, four “fence” pieces (0.60-cm-thick opaque black acrylic) were installed around the clam in individually fitted positions. The two principal fence pieces [solid black in Fig. 1(*b*)] blocked the clam from moving forward or backward. Two additional fence pieces [one depicted with dashed lines in Fig. 1(*b*)] were installed close to the shell laterally, on the two sides.

For measuring pH, we used pH-sensitive foils (SF-HP5R-L4/W4-OIW-US; nominal working range: pH 5-8) manufactured by PreSense Precision Sensing. Foils – ca. 0.3 mm thick when saturated with water – measure 4 cm × 4 cm when at full size. Some clams were large enough that we used foils at this size. For smaller clams, foils were cut to shorter length and/or width. Foils were manufactured with a black optical isolation layer on the upper surface to minimize illumination of the optrode layer by sunlight (this was also the reason that black acrylic was used for the fence pieces). The foil in each aquarium was fastened with one or two thin, marginal strips of black vinyl tape to the acrylic substrate under a clam’s byssal opening [Fig. 1(*b*)].

After set up, each aquarium was fully immersed (24 cm deep) in the outdoor flow-through seawater system at Sesoko Station, resting on a background of black cloth, in a glass-roofed study arena that was freely ventilated with outside air and illuminated only by the sun. Imaging of the pH-sensitive foil was carried out in a nearby, unheated, partly darkened room (fully dark at night), where the study aquarium was carried, full of seawater, as gently as possible. Imaging required ca. 10 min, after which the study aquarium was returned to the seawater system. Images were obtained by use of a PreSens VisiSense detector and analyzed with PreSens AnalytiCal 2 software. Calibration is discussed next.

***Calibration of PreSens foils***

Foil calibration was carried out with seven foil pieces in a PreSens CaliPlate (the foil pieces were from the same manufacturing batch as the foils in study aquaria). The CaliPlate, loaded with foil pieces, was stored underwater next to the clam study aquaria at all times so that calibration foils experienced the same illumination as study foils. For a calibration, the CaliPlate was temporarily removed from the water, and the seven CaliPlate wells with foil pieces were filled with calibration buffer solutions at the same ionic strength (0.72 M) as seawater: pH = 5.69, 5.87, 6.33, 6.96, 7.11, 7.29, and 8.21 (seawater pH). The calibration buffer solutions were prepared from a pH 5.00 potassium hydrogen phthalate buffer (Fisher Scientific SB 102-500), titrated with NaOH, and brought to the appropriate ionic strength with the addition of NaCl. The pHs of the calibration solutions were measured with a Mettler Toledo InLab Routine Pro pH meter. Images of the seven foil pieces in the CaliPlate were obtained (in duplicate) with the VisiSense detector, and a calibration curve was constructed using a sigmoidal fit (Boltzmann equation) as recommended by PreSens (Daniela Obermaier, personal communication).

***Study of pH using fiber-optic microprobes***

Microprobe studies were carried out with PreSens fiber-optic optrodes (PM-HP5-L2.5-NS75/0.5-OIW) mounted in 7.5-cm-long, 21-gauge (0.8 mm OD) dulled hypodermic needles. An optrode of this type measures pH at a single point, its tip (diameter: 0.14 mm). Clams (4.7-7.2 cm long; different individuals from those studied with foils) were set up (without foils) in modified study aquaria that had injection septa installed in the walls at the same vertical height as the byssal margin of the shell [see Fig. 1(*b*)], with modified lateral fence pieces that permitted lateral access to the clam. To measure pH, the hypodermic needle of a probe was inserted into a clam’s seawater-filled aquarium via a septum. Observing with magnifying lenses, we then extended the pH-sensitive tip of the optrode (inside the needle during insertion) so that the optrode tip was fully exposed, positioned in the beveled part of the needle near the needle tip. We then maneuvered the optrode tip (which could be identified exactly by its emission of periodic light pulses) to be at desired locations. Calibration was carried out with the calibration solutions already described.

***Immunofluorescence identification and localization of vacuolar-type H^+^-ATPase (VHA)***

Slices of pedal mantle tissue (ca. 1 cm × 1 cm × 0.5 cm) were vivisected from 5 clams (selected at random from those employed in the foil studies) in Okinawa and immediately placed in 0.1 M sodium cacodylate buffer with 3% paraformaldehyde and 0.35% glutaraldehyde. After 6 h on a shaker plate at 4°C, tissue slices were dehydrated (6 h in 50% ethanol followed by transfer to 70% ethanol). The slices were transported in this state to Scripps Institution of Oceanography. There tissue was embedded in paraffin, sectioned (7 µm), and prepared for immunofluorescence visualization of VHA_B_ following procedures similar to [5].

We first prepared one tissue sample without including borohydrate [6] in the tissue-preparation protocol. The result was excessive background autofluorescence. We thus processed the other 4 tissue samples with borohydrate, and those are the 4 samples for which we report results. The tissue-preparation protocol used for those 4 samples was as follows.

First, sections were rehydrated and permeabilized in 0.2% triton-x in phosphate buffered saline (PBS-T) for 10 min. They were then immersed in sodium borohydrate (1 mg ml^-1^ in PBS-T) six times (10 min each) to quench aldehyde fluorescence [6]. Next, non-specific binding sites were blocked by exposure for 1 h to blocking buffer (2% normal goat serum and 0.02% keyhole limpet hemocyanin in PBS-T, pH 7.8). Following that step, sections were incubated overnight at 4°C with rabbit polyclonal anti-VHA_B_ antibodies (30 μg ml^-1^ in blocking buffer), the primary antibodies. This antibody preparation recognizes the conserved VHA_B_ epitope AREEVPGRRGFPGY and has been used to immunolocalize VHA_B_ in multiple species including not only molluscs [5,7] but also cnidarians [8], annelids [9], and fish [10]. The sections were then incubated with the secondary antibody (goat anti-rabbit Alexa 555, dilution 1:500 in blocking buffer) for 1 h at room temperature, followed by incubation with Hoescht 33342 (Invitrogen, Grand Island, NY, USA; 1 µg ml^-1^ in PBS) for 5 min to visualize nuclei. Between steps, sections were profusely rinsed in PBS. Antibody specificity controls included omission of anti-VHA_B_ antibody, and incubation with anti-VHA_B_ antibodies preabsorbed with 2000-fold excess antigen peptide.

Immunolabeled tissue sections were visualized using a Zeiss AxioObserver Z1 epifluorescence microscope, images of control sections being acquired with the same exposure settings as used for the treatment sections. Digital images of tissue sections were adjusted for brightness and contrast only, using Zeiss Axiovision software. Multiple sections from each of the 4 clam specimens were analyzed.

The image in Figure S1, which can be directly compared with that in Fig. 2(*c*(i)), shows a neighboring control section incubated with anti-VHA_B_ antibodies preabsorbed with excess antigen peptide.


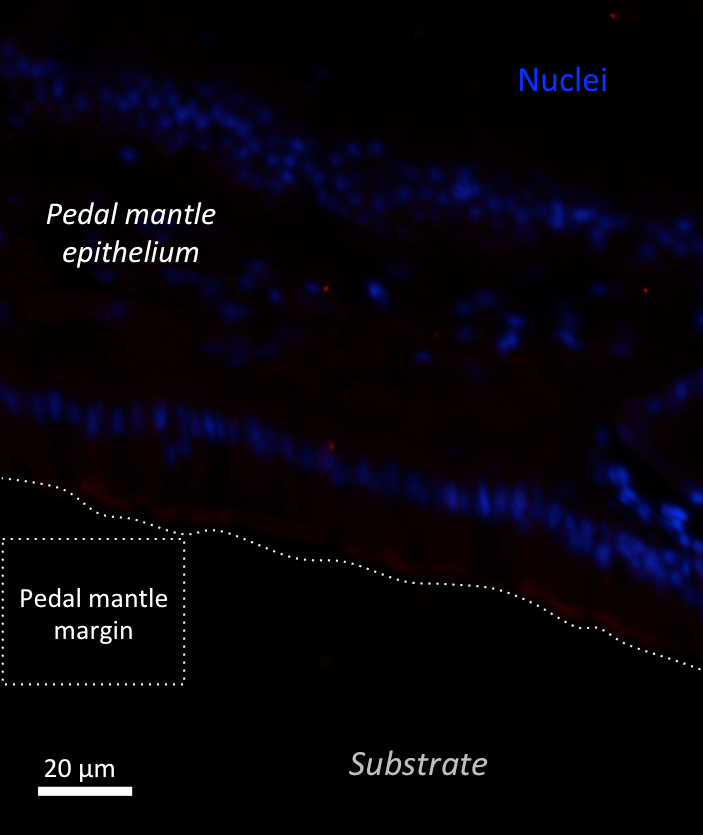


**Figure S1**. Antibody specificity control.  Section of *Tridacna crocea* pedal mantle tissue incubated with anti-VHA_B_ antibodies and 2000-fold excess (mol:mol) blocking peptide and imaged by epifluorescence microscopy.  Cell nuclei are stained blue with Hoescht 33342. The pedal mantle margin (oriented downwards and in contact with the substrate) is delineated by dotted line.

**Supplementary Results**

***Measurement of the response time of foils***

Foil response time was measured by PreSens Precision Sensing (Daniela Obermaier, personal communication) under conditions closely similar to those to which foils were exposed in our study (temperature and ionic strength of buffers used: 19.8°C, 0.7 M). Foils equilibrated in a pH 9 buffer were suddenly moved to a pH 4 buffer (or vice versa), then observed continuously without stirring or shaking. The time to 90% of full response (t_90_) averaged 190 s in going from pH 9 to 4 and 300 s from pH 4 to 9. The results indicate that t_90_ is ≤1 min for a change of 1 pH unit in either direction.

**Literature cited in this ESM**

1. Hedley, C. 1921 A revision of the Australian *Tridacna*. *Rec. Aust. Mus.* **13(4)**, 163–172.

2. Suzuki Y. 1998 Preliminary studies on locomotion and burrowing by juvenile boring clam, *Tridacna crocea*. *Naga* (ICLARM Quarterly) **21(1)**, 31-35.

3. Kawaguti S. 1983 Metamorphosis of the boring clam, *Tridacna crocea*. *Proc. Jpn. Acad. B* **59(4)**, 67-70. (doi: 10.2183/pjab.59.67)

4. Yonge, CM. 1982 Functional morphology and evolution in the Tridacnidae (Mollusca: Bivalvia: Cardiacea). *Rec. Aust. Mus.* **33(17)**, 735–777. (doi: 10.3853/j.0067-1975.33.1981.196)

5. Thomsen J, Himmerkus N, Holland N, Sartoris FJ, Bleich M, Tresguerres M. 2016 Ammonia excretion in mytilid mussels is facilitated by ciliary beating. *J. Exp. Biol.* **219**, 2300–2310. (doi: 10.1242/jeb.139550)

6. Clancy B, Cauller LJ. 1998 Reduction of background autofluorescence in brain sections following immersion in sodium borohydride. *J. Neurosci. Meth.* **83**, 97–102. (doi: 10.1016/S0165-0270(98)00066-1)

7. Barron ME, Roa JNB, Tresguerres M. 2012 Pacific oyster mantle, gill and hemocytes express the bicarbonate-sensing enzyme soluble adenylyl cyclase. *FASEB J.*  **26**, Suppl., 1070.2 (meeting abstract).

8. Barott KL, Venn AA, Perez SO, Tambutté S, Tresguerres M. 2015 Coral host cells acidify symbiotic algal microenvironment to promote photosynthesis. *Proc. Natl. Acad. Sci. USA* **112**, 607–612. (doi: 10.1073/pnas.1413483112)

9. Tresguerres M, Katz S, Rouse GW. 2013 How to get into bones: proton pump and carbonic anhydrase in *Osedax* boneworms. *Proc. R. Soc. B* **280**, 20130625. (doi: 10.1098/rspb.2013.0625)

10. Roa JN, Munévar CL, Tresguerres M. 2014 Feeding induces translocation of vacuolar proton ATPase and pendrin to the membrane of leopard shark (*Triakis semifasciata*) mitochondrion-rich gill cells. *Comp. Biochem. Physiol. A* **174**, 29–37. (doi: 10.1016/j.cbpa.2014.04.003)
